# Colonization by endophytic *Ochrobactrum anthropi* Mn1 promotes growth of Jerusalem artichoke

**DOI:** 10.1111/1751-7915.12145

**Published:** 2014-07-30

**Authors:** Xianfa Meng, Dekai Yan, Xiaohua Long, Changhai Wang, Zhaopu Liu, Zed Rengel

**Affiliations:** 1 Jiangsu Provincial Key Laboratory of Marine Biology, College of Resources and Environmental Sciences, Nanjing Agricultural UniversityNanjing, 210095, China; 2 Soil Science and Plant Nutrition, School of Earth and Environment, The University of Western AustraliaCrawley, WA, 6009, Australia

## Abstract

The *Ochrobactrum anthropi* Mn1 strain, taxonomically identified using 16S ribosomal DNA sequence, was isolated from roots of Jerusalem artichoke. Its endophytic colonization was investigated microscopically using green fluorescent protein introduced by vector pHC60. The strain entered Jerusalem artichoke tissues through the root, and was localized in the roots and stems. The plant growth-promoting (PGP) effects of *O*. *anthropi* Mn1 were assessed in greenhouse as well as field trials with different nitrogen supplies. Only under moderate to ample nitrogen supply, could *O*. *anthropi* Mn1 promoted growth of host plant. The PGP effects of the strain were symbiotic nitrogen fixation, root morphological optimization and enhanced nutrient uptake. We hypothesize that the symbiotic interspecies interaction might be quorum sensing related.

## Introduction

For centuries, plant growth-promoting bacteria (PGPB) have been used in agricultural practices mainly as bioferilizers (Sturz *et al*., 2000; Weyens *et al*., 1965); such bacteria can enhance plant resistance to environmental stresses (Shen, 1997). PGPB were defined as microorganisms that possess the capacity to promote growth and/or suppress disease when present in the rhizosphere and as endophytes within healthy plant tissues (Conn and Franco, 2004). In the rhizosphere, PGPB may colonize soil that immediately surrounds roots (Rovira, 1991) or attach to the root surface (Andrews and Harris, 2000). Isolated from surface-disinfested plant tissues, endophytes are those microbes that do not harm plants (Hallmann *et al*., 1997). A range of endophytic bacteria isolated from various tissues of cereals and grasses, including *Azotobacter* (Zahir *et al*., 2003), *Azospirillum* (Thakuria *et al*., 2004), *Bacillus* (Çakmakçı *et al*., 2007), *Burkholderia* (Kennedy *et al*., 2004), *Klebsiella* (El-Khawas and Adachi, 1999) and *Pseudomonas* (Beneduzi *et al*., 2008), were found to promote plant growth.

Many members of alphaproteobacteria are capable of interacting with eukaryotic cells and functioning as endophytic symbionts. Some of them (including well-known genera *Rhizobacteria*, *Agrobacterium*, *Rickettsia*, *Bartonella* and *Brucella*) could fix nitrogen and are thus agriculturally important (Ugalde, 1999; Batut *et al*., 2004). *Ochrobactrum anthropi* is a Gram-negative bacterium from the family *Brucellaceae* (alphaproteobacterial order *Rhizobiales*); it is genetically and phenotypically diverse in various habitats including soil, plants and their rhizosphere, animals and humans (Bathe *et al*., 2005). Regarding plant associations, *O. anthropi* was mostly isolated from the rhizosphere or the rhizoplane. An isolate of *O. anthropi* (TRS-2) was suggested to (i) facilitate plant nutrient uptake from soil and (ii) prevent plant diseases (Chakraborty *et al*., 2009).

Similar to rhizosphere PGPB, endophytic bacteria may elicit plant growth promotion mainly via nitrogen fixation, phosphate solubilization, indole 3-acetic acid (IAA) and/or siderophore production (Baltruschat *et al*., 2008; Franche *et al*., 2009; Long *et al*., 2010,2010). Jerusalem artichoke (*Helianthus tuberosus* L.) is an agricultural and industrial crop with a great potential for food, production of ethanol and industrial products (Long *et al*., 2010,2010; Jin *et al*., 2013). Isolated from roots of Jerusalem artichoke in the previous work (Meng *et al*., 2011), *O. anthropi* Mn1 was characterized in this study for plant growth promotion as endophytic N_2_-fixing bacteria. The relationship between N supplementation and effectiveness of *O. anthropi* Mn1 *in vivo* was elucidated.

## Results

### Identification and characterization of isolate Mn1

**Fig 1:**
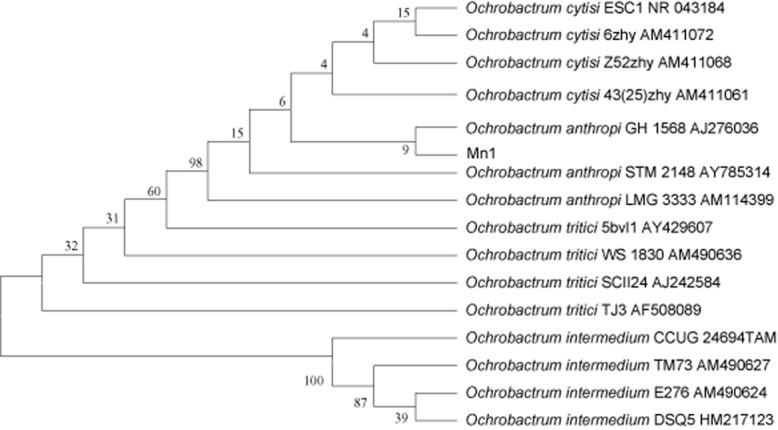
Phylogenetic tree showing genetic relationship of *O*. *anthropi* Mn1 with taxonomically similar strains of *Ochrobactrum* available in the GenBank database (accession numbers are in parentheses) based on 16S rDNA sequence. Numbers on branches represent the percentage bootstrap support calculated for 1000 replicates.

Isolated from the Jerusalem artichoke roots, strain Mn1 was an endophytic N_2_-fixing bacteria (Meng *et al*., 2011). On the basis of 16S rDNA sequence, the isolate was identified as *O. anthropi*. Using the sequences of 16S rDNA of *Ochrobactrum* available in the GenBank database, a phylogenetic tree was constructed based on per cent divergence after alignment with the Clustal W method (Fig. 1). Isolate Mn1 had the highest similarity to *O. anthropi* GH 1568 belonging to the *O. anthropi* monophyletic group and was designated *O. anthropi* Mn1. The properties of *O. anthropi* Mn1 related to plant growth promotion were determined, including nitrogen fixation, phosphate solubilization and IAA and siderophore production (Table 1).

**Table 1:** Plant growth-promotion properties of *O*. *anthropi* Mn1

Strain	Nitrogenase activity (nmol ml^–1^·h)	IAA production (μg ml^–1^)	Siderophore production (λ λ_0_^–1^)	Phosphate solubilization (μg ml^–1^)
*O. anthropi* Mn1	207.34 ± 15.1	55.49 ± 4.27	0.35	6.99 ± 0.71
*O. anthropi* Mn1g	207.58 ± 8.62	55.40 ± 6.70	0.36	6.91 ± 0.62

The ± standard error of each mean (*n* = 3) is indicated in the figure. Siderophore production: little, 0.8–1.0; low, 0.6–0.8; moderate, 0.4–0.6; high, 0.2–0.4; very high, 0–0.2.

### Colonization of plant tissues by *O*. *anthropi* Mn1

**Fig 2:**
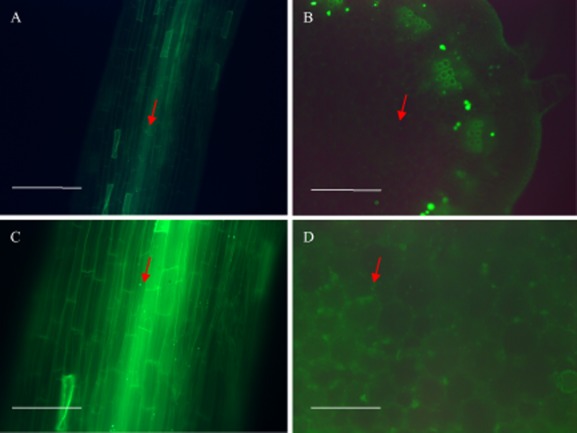
Fluorescence micrographs showing the colonization of roots and stems of micropropagated Jerusalem artichoke seedlings by *O*. *anthropi* Mn1g. Arrows indicate visualized bacteria. (A, C) Endophytic colonization of tissues of surface-sterilized root at 15 DAI. (B, D) Endophytic colonization of surface-sterilized stem at 15 DAI. (A, B) Bar = 10 μm; (C, D) bar = 20 μm.

To examine endophytic colonization of Jerusalem artichoke seedlings by *O. anthropi* Mn1, the *gfp* gene was introduced into the bacteria with plasmid pHC60 by triparental mating. *Ochrobactrum anthropi* Mn1g exhibited strong green fluorescent property, and had the same growth curve in nutrient medium and the plant growth-promoting (PGP) properties as the parent *O. anthropi* Mn1 after subculture (Table 1). *Ochrobactrum anthropi* Mn1g was found to colonize roots and stems (but not leaves) of Jerusalem artichoke seedlings at 15 days after inoculation (DAI) (Fig. 2).

**Fig 3:**
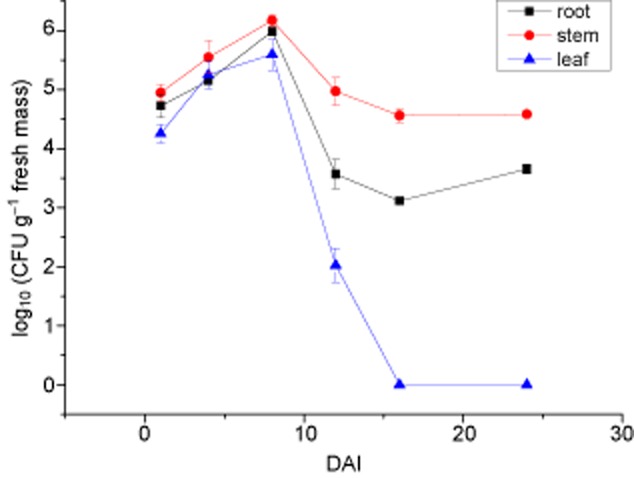
Endophytic population in Jerusalem artichoke tissues after seedlings were inoculated with *O*. *anthropi* Mn1g. The ± standard error of each mean (*n* = 3) is indicated in the figure.

*Ochrobactrum anthropi* Mn1g was re-isolated from tissues, indicating internal colonization and spreading in the plants. To define the population dynamic of *O. anthropi* Mn1g in Jerusalem artichoke plants, roots, stems and leaves were plated (Fig. 3). In root and stem tissues, there was a transient burst of endophyte growth followed by a decline and then a levelling off. In leaves, population of *O. anthropi* Mn1g started to decline at 8 DAI, and no bacteria was detected at 16 DAI. The fact that *O. anthropi* Mn1g could be re-isolated from stem and leaf tissues clearly indicated that the strain spread from roots to other seedling parts. The population of *O. anthropi* Mn1g in tissues of plant showed that its growth was best in stem (Fig. 3).

### PGP effects of *O*. *anthropi* Mn1

**Fig 4:**
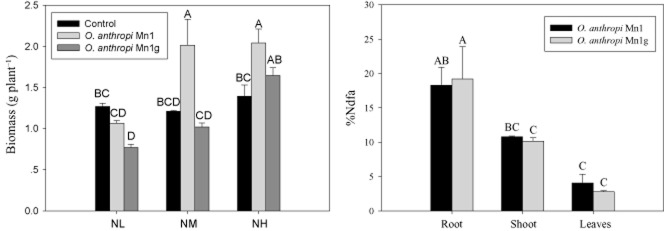
Growth-promoting effects of *O*. *anthropi* Mn1 and *O*. *anthropi* Mn1g with differential nitrogen supply (left) and N-fixing contribution in various tissues with medium nitrogen supply (right) at 40 DAI. NL, NM and NH stand for low, medium and high nitrogen level with (NH_4_)_2_SO_4_ supply of 0, 2 and 10 mmol l^−1^ respectively. %Ndfa, percentage of nitrogen derived from air, referring to the amount of nitrogen transferred to plant tissues via BNF as measured using ^15^N isotope dilution method.

Analysis carried out at 40 DAI showed that the strain had a differential effect on Jerusalem artichoke plants under various nitrogen supplies (Fig. 4). Without nitrogen input, biomass of seedlings inoculated with *O. anthropi* Mn1 and particularly *O. anthropi* Mn1g was lower than the control. In contrast, inoculation of *O. anthropi* Mn1 made a significant increase in biomass with moderate and high nitrogen supplement. Only under high nitrogen supplement could *O. anthropi* Mn1g enhanced plant dry matter, as it needed more energy to generate green fluorescent protein.

Biological nitrogen fixation (BNF) in Jerusalem artichoke plants by endophytic bacteria at 40 DAI was estimated using ^15^N isotope dilution (Fig. 4). Both *O. anthropi* Mn1 and *O. anthropi* Mn1g contributed nitrogen to host plants through BNF to a similar extent. The highest BNF contribution was measured in roots followed by stems and leaves.

Under greenhouse conditions, nitrogen fertilization had different effects on root properties with or without endophytic diazotroph colonization 40 DAI (Table 2). The root properties of plants inoculated with either *O. anthropi* Mn1 or Mn1g were the highest under medium nitrogen treatment, whereas non-inoculated plants showed the best root properties under low N fertilization. Endophytic bacteria stimulated Jerusalem artichoke root growth at medium and high nitrogen fertilization when compared with plants without inoculation.

**Table 2:** Effect of endophytic bacteria (with or without *O*. *anthropi* Mn1 or *O*. *anthropi* Mn1g) and nitrogen fertilization on the root length, surface area, volume and number of root tips at 40 DAI

	CK × NL	CK × NM	CK × NH	Mn1 × NL	Mn1 × NM	Mn1 × NH	Mn1g × NL	Mn1g × NM	Mn1g × NH
Root length (cm)	273c	193cd	221cd	285c	410b	320bc	228d	550a	299bc
Surface area (cm^2^)	55c	42cd	49cd	55c	87b	63bc	25d	124a	64bc
Root volume (cm^3^)	0.9c	0.7cd	0.9cd	0.9cd	1.5b	1.0c	0.4d	2.2a	1.1bc
Number of root tips	290ab	231ab	291ab	271ab	496ab	462ab	198b	527a	490ab

CK, Mn1 and Mn1g: without inoculation, inoculation with *O. anthropi* Mn1 and *O. anthropi* Mn1g respectively. NL, NM and NH stand for low, medium and high nitrogen level with (NH_4_)_2_SO_4_ supply of 0, 2 and 10 mmol l^–1^ respectively. The test was significant at the 5% level (*P* ≤ 0.05) and shown with letters a, b, c and d.

**Fig 5:**
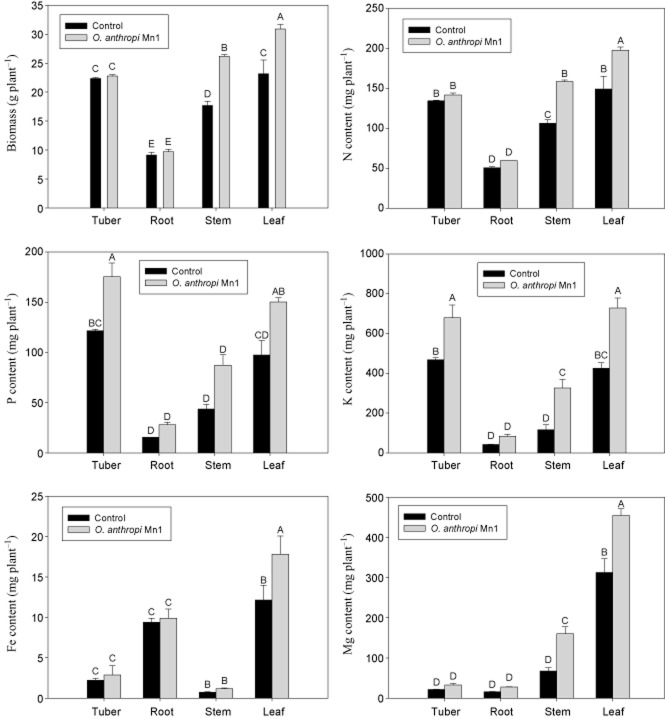
Effect of *O*. *anthropi* Mn1 on biomass and nutrient content of Jerusalem artichoke under field trial on arenaceous soil without fertilizer (2 years pooled data).

In a 2 year field trial, plants inoculated with *O. anthropi* Mn1, which had been re-isolated afterwards for confirmation, showed an increase in dry weight and nutrient content compared with the plants without inoculation (Fig. 5). *Ochrobactrum anthropi* Mn1 inoculation increased biomass of stems and leaves significantly. Tissue nitrogen concentration (especially in stems and leaves) was significantly higher with than without bacterial inoculation. Endophytic bacteria could also promote uptake of P, K, Fe and Mg by Jerusalem artichoke, with significant differences observed in concentration of these nutrients in stems and leaves.

## Discussion

The alphaproteobacterial genus *Ochrobactrum*, first described by Holmes and colleagues (1988), currently comprises five described species, *O. anthropi*, *O. intermedium*, *O. tritici*, *O. grignonense* and *O. gallinifaecis* (Lebuhn *et al*., 2006). Associated with eukaryotic cells, members of *Ochrobactrum* species play important roles as intracellular symbionts or pathogens in plants, animals and humans (Graham *et al*., 2007). Recent reports have described isolates of *Ochrobactrum* that could enhance growth of mung bean (Faisal and Hasnain, 2006), tea (Chakraborty *et al*., 2009), *Acacia mangium* (Ngom *et al*., 2004) and cucumber (Zhao *et al*., 2012) via assisting nutrient uptake, engaging in symbiotic nitrogen fixation and preventing plant diseases. *Ochrobactrum anthropi*, used to be recognized as opportunistic pathogen (Teyssier *et al*., 2005), constitutes up to 2% of the culturable bacteria from soil (Lebuhn *et al*., 2006). The tea rhizobacterium *O. anthropi* TRS-2 solubilized phosphate produced siderophore and IAA *in vitro* and had antagonistic activity against pathogens (Chakraborty *et al*., 2009). Isolated from banana leaf, endophytic bacterium BA131 was phylogenetically close to *O. anthropi* (Cruz *et al*., 2001). In our study, strain Mn1, previously isolated from the root of Jerusalem artichoke as endophytic diazotroph, was identified as *O. anthropi* according to the phylogenetic analysis of 16S rDNA (Fig. 1).

Fluorescent protein genes have been widely used to study plant–microbe interactions because they provide fast real-time temporal resolution (Maciá-Vicente *et al*., 2009). To assess the localization and enumerate *O. anthropi* Mn1, vector pHC60 with *gfp* gene was employed (Fig. 2), but the method may not be suitable for leaves because chloroplasts may mask the green fluorescence (Njoloma *et al*., 2009). Colonized xylem vessels of host plants is the main route for endophytic bacteria to move from the roots to the aerial parts (James and Olivares, 1998; James *et al*., 2000). Alternative opinions also exist (Dong *et al*., 1997). In this study, micropropagated Jerusalem artichoke plants with open-entry points at the base of the stem were easy for endophytic bacteria to enter. After infection, bacteria entered vascular tissues, and colonized intercellular spaces of stem and leaf tissues. *Ochrobactrum anthropi* Mn1g apparently was inclined to colonize in stem tissues, and consequently a higher abundance was detected than in the root or leaf tissues (Fig. 3). *Ochrobactrum anthropi* Mn1g multiplied as the host plant grew until a balance was achieved between plant and endophyte (Nogueira *et al*., 2001), which might have happened around day 8 after inoculation (Fig. 3). Generally, plants may develop oxidative nitrogen scavenging (ONS) strategy by secreting reactive oxygen species to oxidize symbiotic diazotrophic bacteria and extract nitrogen from them (White *et al*., 2012). This might be a reason for the decreased of bacteria in root, stem and leaf tissues in the present study at 8 DAI (Fig. 3). Sixteen DAI, *O. anthropi* Mn1g populations in root and stem tissues reached stationary phase (Fig. 3). However, in leaf it dropped to zero by day 16 after inoculation (Fig. 3). We could not explain the influence of plant age on the population decrease. However, since the decline appeared when the population reached analogous climax, similar quorum-sensing mechanisms might be present as in the report by Dourado and colleagues (2013).

Endophytic bacteria may promote plant growth through nitrogen fixation (Gyaneshwar *et al*., 2002; Paungfoo-Lonhienne *et al*., 2014), phytohormone production (Chen *et al*., 2010; Malfanova *et al*., 2011) and enhanced uptake of mineral nutrients (Rajendran *et al*., 2008; Weyens *et al*., 2013). In our study, the *O. anthropi* Mn1 strain fixed nitrogen in association with the host plant when colonized in root and stem tissues. The percentage of biologically fixed nitrogen was higher in root than stem or leaf tissues (Fig. 4), even though abundance of endophytic diazotrophic bacteria was lower in root than stem tissues (Fig. 3). This discrepancy might have arisen from some bacteria localized at the root surface fixing nitrogen, but not being counted because plant tissues were surface sterilized before being used for bacteria enumeration. The diazotrophic bacteria may be digested by the host plant through the mechanism of ONS (White *et al*., 2012). This would explain relatively low percentage of plant N derived from N_2_ fixation (%Ndfa) detected in leaf tissues (Fig. 4) because bacteria had the presence there initially, but disappeared from day 16 (Fig. 3).

Relatively high concentration of IAA was detected in the secretion of *O. anthropi* Mn1 (Table 1). IAA is known to stimulate both rapid and long-term growth responses in plants (Cleland, 1971) and could increase the plant biomass. A low concentration of IAA promotes primary root elongation, whereas a high IAA concentration stimulates lateral and adventitious root formation (Patten and Glick, 2002). As a result of inoculation with *O. anthropi* Mn1, the root morphology of Jerusalem artichoke changed, including root number, length, volume and surface area (Table 2), which would lead to higher nutrient uptake in the field trial (Fig. 5).

*Ochrobactrum anthropi* Mn1 produced siderophores (Table 1) that are involved in Fe chelation and uptake. Iron taken up by bacteria could have been made available to the host plant following the death of bacterial cells via oxidative scavenging. Hence, the endophytic bacteria *O. anthropi* Mn1 rely on supply of some nutrients by the host plant and can in return supply the host plants with other nutrients (e.g. N and Fe). However, a good external supply of N is required for bacteria to get established and start to fix atmospheric N, eliciting a growth-promoting effect (Fig. 4).

## Experimental procedures

### Isolation and identification of *O*. *anthropi* Mn1

*Ochrobactrum anthropi* Mn1 was isolated from the root of Jerusalem artichoke (Meng *et al*., 2011). After surface sterilization, root tissue was macerated using a sterile mortar and pestle. Samples of tissue extracts and their different dilutions were plated onto Ashby's nitrogen-free agar medium. Bacterial cells sampled from the colonies after incubation at 28°C for 48 h were streaked for purification on lysogeny broth (LB) agar medium.

*Ochrobactrum anthropi* Mn1 was selected to have high nitrogenase activity (see the next paragraph) and identified by determination of 16S rDNA sequences. Colony polymerase chain reaction was performed to generate a 1200 bp portion of the 16S rDNA using the primers 27f (5-AGAGTTTGATCCTGGCTCAG-3) and 1492r (5-TACGGCTACCTT GTTACGACTT-3) (Byers *et al*., 2006). The partial 16S rDNA sequence was obtained from Beijing Genomics Institute (Beijing, China) and compared with sequences in GenBank database using the NCBI blast program. Its phylogenetic tree was generated using mega version 4.0 (Tamura *et al*., 2007) with the sequences of 16S rDNA and the related sequences obtained by blast.

### Determination of PGP properties of *O*. *anthropi* Mn1 *in vitro*

To test the N_2_-fixing activity of *O. anthropi* Mn1, an acetylene reduction assay was used (Meng *et al*., 2011). The strain was grown in 5 ml cotton-plugged culture vials containing 2 ml of Ashby's nitrogen-free semi-solid medium at 30°C for 48 h. Cotton plugs were aseptically exchanged with rubber stoppers, and the headspace air was replaced with 10% (by volume) of high purity C_2_H_2_ gas using a hypodermic syringe. The ethylene (C_2_H_4_) production was determined after 48 h of incubation in the dark at 30°C. Vials without C_2_H_2_ served as controls.

IAA production was measured according to the method of Amaresan and colleagues (2012). *Ochrobactrum anthropi* Mn1 was inoculated in nutrient broth containing tryptophan (0.5 mg ml^−1^) and incubated at 30°C for 5 days. After centrifugation, supernatant (2 ml) was mixed vigorously with 4 ml of Salkowski's reagent and two drops of orthophosphoric acid. After 20 min incubation at room temperature, IAA production was assessed by measuring absorbance at 530 nm using a spectrophotometer. The IAA concentration was determined using a calibration curve of pure IAA as a standard following the linear regression analysis.

Production of siderophores was quantified using tertiary complex chromazurol-S (CAS)/Fe^3+^/hexadecyl trimethyl ammonium bromide as an indicator. The reaction was carried out as described elsewhere (Schwyn and Neilands, 1987). Bacterial supernatant was obtained after culturing in King B medium without phosphate and was mixed with CAS assay solution in the equal volumes. After 3 h incubation in the dark at 30°C, the absorbance at 630 nm was recorded with non-inoculated supernatant as reference.

The strain was analysed for inorganic phosphate solubilization using the ascorbic acid method (Watanabe and Olsen, 1965). A loopful of fresh bacterial culture was grown in Pikovskaya broth at 30°C for 10 days. The soluble phosphorus in the culture filtrate was mixed with Mo-solution. With uninoculated broth serving as control, the absorbance of the mixture was measured on a spectrophotometer at 730 nm to assess the quantity of solubilised phosphate.

### Localization and colonization of *O*. *anthropi* Mn1 *in vivo*

The plasmid vector pHC60, which is resistant to tetracycline and could constitutively express the *gfp* gene, was transferred to the wild-type strain of *O. anthropi* Mn1 using the triparental mating method (Cheng and Walker, 1998). *Escherichia coli* DH5α donor strain (harbouring plasmid pHC60) and HB101 helper strain (harbouring plasmid pRK2013 containing *tra* gene) (both strains are resistant to kanamycin) were grown overnight in LB broth (pH 7.0) at 37°C. *Ochrobactrum anthropi* Mn1 was grown on LB broth containing 50 mg ml^−1^ ampicillin and used as a recipient strain. Cell suspensions from donor, helper and recipient strains were mixed in a 3:3:1 volumetric ratio, and the mixture was plated on LB agar at 30°C. The following day, a loopful of bacteria was streaked onto LB plates containing kanamycin (100 mg ml^−1^), ampicillin (50 mg ml^−1^) and tetracycline (50 mg ml^−1^) for selection. The positive colony that expressed *gfp* gene stably was obtained after subculturing 30 generations with and without selective pressure, and was named *O. anthropi* Mn1g.

Micropropagated Jerusalem artichoke plants were obtained according to the method developed by El Mostafa and colleagues (2008). Leaves of Jerusalem artichoke were rinsed in sterile water for 20 min and then surface sterilized following the procedure described above. After rinsing three times in sterile distilled water, leaf explants were placed in Petri dishes containing Murashige–Skoog basal medium supplemented with 1-Naphthaleneacetic acid (0.1 mg l^−1^). Cultures were grown at 16/8 h photoperiod (light/dark) and 25 ± 1°C for 5–6 weeks. The germinated seedlings were aseptically transferred to glass tubes (4 cm in diameter, 29 cm in height) containing sterilized vermiculite saturated with half-strength sterile Hoagland nutrient solution (Hoagland and Arnon, 1950). Seedlings were cultured for 3 days at 25 ± 1°C, then the roots were inoculated with *O. anthropi* Mn1g by dipping into bacterial suspension containing 10^5^–10^6^ bacteria ml^−1^ for about 30 min [the root system was reported to be the main entry avenue for endophytic bacteria (Hurek *et al*., 1994)]. The roots were rinsed in sterile water to remove loosely adhering bacteria, and the seedlings were grown in sterile vermiculite. Non-inoculated plants were used as negative controls.

### Fluorescence microscopy

Fifteen DAI, seedlings were surface sterilized and then separated into roots, stems and leaves. The knife used was washed in 70% v/v alcohol and sterile water to avoid cross contamination of tissues during excision. To examine the localization of the inoculated bacteria in tissues, freehand section was used for slide preparation in a sterile airflow cabinet. These sections were examined for green fluorescence from *gfp*-tagged bacteria using automated Leica DM5000 B microscope (Leica Microsystems, Cambridge, UK).

### Enumeration of bacteria

The seedlings inoculated with *O. anthropi* Mn1g (see 2.3.2) were harvested and sampled at 1 and 8 h, and then after 1, 4, 8, 12, 16 and 24 DAI to determine bacterial population. After surface sterilization, roots, stems and leaves were excised, weighed and ground in a mortar and pestle in 0.8% w/v saline solution. The macerate was serially diluted and plated on LB agar plates containing tetracycline (50 mg ml^−1^). Using an Olympus SZX12 fluorescence stereo microscope, *gfp*-tagged and wild-type bacterial colonies (with and without green fluorescence, respectively) were counted on the plates after incubation at 30°C for 2 days.

### N_2_-fixing capacity of bacteria

^15^N isotope dilution method was used to define the role of biological N_2_ fixation in the N economy of the endophyte-plant system. Micropropagated Jerusalem artichoke plants were inoculated with *O. anthropi* Mn1 as well as *O. anthropi* Mn1g (non-inoculated plants were used as controls). The Fahraeus N-free liquid plant growth medium supplemented with (NH_4_)_2_SO_4_ (11.7 atom per cent ^15^N excess) at concentration of 2 mmol l^−1^ (Fahraeus, 1957) was used for growing plants in a greenhouse at 16/8 h photoperiod (light/dark) and 25 ± 1°C for 40 days. Roots, stems and leaves were separated, washed, dried at 70°C and ground to fine powder. Samples of plants were assayed for ^15^N content and total N using Flash-2000 coupled with Delta V ADVANTAGE (Thermo Fisher Scientific, Waltham, MA, USA). %Ndfa by *O. anthropi* Mn1 was calculated as follows: %Ndfa = [1 − (^15^N atom % excess inoculated plant/^15^N atom % excess control plant)] × 100 (Oliveira *et al*., 2002).

### Growth-promoting effect of *O*. *anthropi* Mn1

Under greenhouse conditions [16/8 h photoperiod (light/dark) and 25 ± 1°C], micropropagated Jerusalem artichoke plants were inoculated with *O. anthropi* Mn1 or *O. anthropi* Mn1g. Plant growth was supported by the Fahraeus N-free liquid medium supplemented with different (NH_4_)_2_SO_4_ concentrations (0, 2 or 10 mmol l^−1^). Forty DAI, Jerusalem artichoke plants were carefully washed in tap water to remove the vermiculite from the roots. Seedlings were separated into roots, stems and leaves. A subsample of roots was used to determine the root-related parameters (length, surface area, volume and number of tips) by a scanner (LA1600 + scanner, Canada) followed by analyses by Win-Rhizo software (Win-Rhizo 2003b, Canada). The remaining roots as well as stems and leaves were oven-dried at 70°C and weighed.

A 2-year field experiment (2011–2012) was conducted to study the effect of *O. anthropi* Mn1 on growth and nutrient uptake of Jerusalem artichoke at Laizhou, Shandong, China, located at 37°38′N and 119°38′E on arenaceous soil without fertilizer (Meng *et al*., 2012). In April, micropropagated Jerusalem artichoke plants were inoculated with the endophytic diazotrophic strain (control plants were dipped into sterile water) and transplanted into soil. Seven months later, whole plants were collected, and the soil was washed off. Tubers, roots, stems and leaves were separated before drying at 70°C. Re-isolation of inoculated strain was done as described above. The dried samples were weighed, ground thoroughly to pass through mesh number 20 and wet digested with concentrated H_2_SO_4_ for determination of total N, P, K, Ca and Mg. A Kjeltec autoanalyser employed Kjeldahl method to determine the N concentration (Heldrich, 1990). Concentrations of P, K, Ca and Mg were determined using inductively coupled plasma optical emission spectrometry.

### Statistical analyses

All experiments in the present study were done in at least three replicates. Analysis of variance was done, and data means were compared using Duncan's multiple range test with spss statistical software (IBM). A probability of *P* ≤ 0.05 was considered significant unless stated otherwise.
